# Tuberculosis incidence rate time series in the state of Santa Catarina, Brazil: analysis of a decade, 2010-2019

**DOI:** 10.1590/S2237-96222022000300002

**Published:** 2022-09-05

**Authors:** Andrielly Pereira, Danúbia Hillesheim, Fábio May da Silva, Regina Célia Santos Valim, Ana Luiza Curi Hallal

**Affiliations:** 1 Curso de Medicina, Universidade Federal de Santa Catarina, Florianópolis, SC, Brazil; 2 Programa de Pós-Graduação em Saúde Coletiva, Universidade Federal de Santa Catarina, Florianópolis, SC, Brazil; 3 Departamento de Cirurgia, Universidade Federal de Santa Catarina, Florianópolis, SC, Brazil; 4 Departamento de Clínica Médica, Universidade Federal de Santa Catarina, Florianópolis, SC, Brazil

**Keywords:** Tuberculosis, Epidemiology, Regression Analysis, Incidence, Time Series Studies

## Abstract

**Objective:**

To describe the profile of tuberculosis cases and analyze the temporal trend of tuberculosis incidence rate in Santa Catarina, by sex, from 2010 to 2019.

**Methods:**

This was a time series study conducted using data from the Notifiable Health Conditions Information System (SINAN). The Prais-Winsten regression model was used.

**Results:**

There were 16,446 new cases of tuberculosis. Most cases occurred in males (68.5%), people aged 20 to 49 years (70.3%), in Greater Florianópolis (25.1%) and in individuals with incomplete elementary education (40.0%). A falling trend in tuberculosis incidence rates was found for males (APC: -1.86%; 95%CI -2.68;-1.03), females (APC: -1.92%; 95%CI -2.63;-1.20) and both sexes (APC: -1.77%; 95%CI -2.37;-1.17).

**Conclusion:**

In the decade analyzed, there was a significant reduction in the tuberculosis incidence rate in Santa Catarina, in both sexes. There was a predominance of males, people of economically active age and with low schooling.

**Study contributions**

**Main results**

Between 2010 and 2019, there was a significant reduction in the tuberculosis incidence rate in both sexes. A greater proportion of cases occurred in males, in Greater Florianópolis, in individuals aged 20 to 49 years and with incomplete elementary education.

**Implications for services**

Despite the decrease found, the need remains to strengthen actions to address the disease in priority populations and to improve public policies that address the social determination of the disease.

**Perspectives**

Finding strategies to reduce tuberculosis incidence in the state, especially among priority groups and in regions with higher population density. In addition, we highlight the importance of the increasing improvement of data records held on the SINAN system.

## INTRODUCTION

Tuberculosis is one of the major health issues to be addressed globally.^1^ ^,^ ^2^ ^^It affects males, adults and people in low-income countries to a greater extent, indicating a link between the occurrence of tuberculosis and socioeconomic factors.^3^ Although measures for tuberculosis prevention and control exist, they do not reach the entire population equally, mainly due to insufficient funding and political involvement.^4^

In 2020, there were 9.9 million new cases worldwide, equivalent to 127 cases per 100,000 inhabitants.^5^ In the same year, 66,819 cases of tuberculosis were diagnosed in Brazil, corresponding to an incidence rate of 31.6 cases/100,000 inhab.^6^ Tuberculosis incidence in Brazil can be considered low, when compared to African and Southeast Asian countries (> 100 cases/100,000 inhab.).^5^ However, 57 countries, located mainly in the Eastern Mediterranean, Europe and North America, had low tuberculosis incidence (< 10 cases/100,000 inhab.) in 2020.^5^

Santa Catarina was among the eight Brazilian states with the lowest incidence rate (16.9/100,000 inhab.), and one of the four with the lowest mortality rate (1.1/100,000 inhab.) in 2020.^6^ A study conducted in Santa Catarina between 2002 and 2009 found a significant reduction (0.9% per year) in tuberculosis incidence.^7^ However, no time series studies were carried out after that period, aimed at monitoring new cases and the profile of those affected. Knowing the epidemiological scenario is necessary for the formulation of effective public policies, directed toward controlling and monitoring infection. 

Given this context, the objective of the study was to describe the profile of tuberculosis cases and analyze the temporal trend of tuberculosis incidence rate in Santa Catarina, Brazil, by sex, from 2010 to 2019.

## METHODS

This was a descriptive time-series study of tuberculosis incidence, conducted using data from the Ministry of Health Notifiable Health Conditions Information System (SINAN) and data from the Brazilian Institute of Geography and Statistics (IBGE), obtained from the Brazilian National Health System Department of Information Technology (DATASUS) website on June 10, 2021.

We analyzed the cases reported in the state of Santa Catarina from 2010 to 2019, including only new and confirmed cases of tuberculosis in adults (≥ 20 years). Ministry of Health guidelines state that new cases refer to people with tuberculosis registered on the SINAN under the following options: new case; not known; and post-death.^6^ ^,^ ^8^

The following variables were analyzed: ‘sex’ (male; female), year of notification (between 2010 and 2019), age group (in years: 20-29; 30-39; 40-49; 50-59; 60-69; 70-79; 80 or over), clinical form of tuberculosis (pulmonary; extrapulmonary; pulmonary and extrapulmonary), Santa Catarina health macro-region (Planalto Norte e Nordeste; Grande Oeste; Meio Oeste e Serra Catarinense; Foz do Rio Itajaí; Alto Vale do Itajaí; Grande Florianópolis; Sul) and schooling (illiterate; incomplete elementary education; complete elementary education; incomplete high school education; complete high school education; incomplete higher education; complete higher education). 

The crude incidence rates were calculated by dividing the number of new cases in the study population by the number of inhabitants estimated by the IBGE for the same period, multiplied by 100,000 inhabitants. In order to avoid the effect of age differences in the population over the years and to enable comparison, we standardized the tuberculosis rates by age, adopting the direct method. We used the standard population of Brazil as estimated by the 2010 demographic census.^9^

The Prais-Winsten regression model was used to analyze trends.^10^ This model acts to correct for the so-called first-order autocorrelation effect, often found in population data metrics.^10^ In this study in particular presence of autocorrelation was assessed using the Durbin-Watson hypothesis test and autocorrelation and partial autocorrelation plots of the time series (not shown). The dependent variable was the logarithm of the standardized incidence rates, and the independent variable was comprised of the years of the time series. We applied the formula proposed by Antunes & Cardoso^10^ to verify annual percent change (APC). APC and the 95% confidence intervals (95%CI) were obtained using the following formulae:

APC = [-1 + 10^b] *100%

95%CI = [-1+10^(b ± t * SE)] *100%

Where: the value of *b* and the standard error (SE) are extracted from the regression; and the value of t is given by the Student’s *t* probability distribution table, with a 95% confidence level. Therefore, in the case of hypothesis testing in H_0_, a stable trend is assumed, and if H_0_ is not rejected (p-value ≥ 0.05), this indicates a stable trend, while if H_0_ is rejected (p-value < 0.05), this indicates a rising or falling trend, depending on whether the change is positive or negative. Data tabulation was initially performed using Microsoft Office Excel 2019®; the data were later exported and analyzed using Stata 14 statistical software. 

As public domain data with no identification of the participants were used, the study project did not need to be submitted for assessment by a Research Ethics Committee.

## RESULTS

A total of 16,446 new tuberculosis cases were notified in the state of Santa Catarina. Comparatively, the highest proportion of cases occurred in males (68.5%), individuals aged 20 to 49 years (70.3%), and in the Greater Florianópolis health macro-region (25.1%). The clinical form of the majority of cases was confirmed as pulmonary (78.8%) and individuals with incomplete elementary school education accounted for the highest share by age group (40.0%) (Table 1).

**Table 1:** - Tuberculosis case distribution according to sociodemographic characteristics and clinical form of the disease, in individuals aged ≥ 20 years, Santa Catarina, 2010-2019

Variables	n	%
Sex		
Male	11,273	68.5
Female	5,173	31.5
Age group (in years)		
20-29	4,095	24.9
30-39	4,063	24.7
40-49	3,417	20.7
50-59	2,673	16.2
60-69	1,418	8.6
70-79	572	3.4
≥ 80	208	1.5
Schooling^a^		
Illiterate	387	2.8
Incomplete elementary education	5,498	40.0
Complete elementary education	3,199	23.2
Incomplete high school education	1,080	7.8
Complete high school education	2,401	17.4
Incomplete higher education	432	3.1
Complete higher education	756	5.7
Clinical form of tuberculosis^a^		
Pulmonary	12,967	78.8
Extrapulmonary	2,698	16.4
Pulmonary and extrapulmonary	779	4.8
Santa Catarina Health Macro-Region^a^		
Sul	2,431	14.8
Planalto Norte e Nordeste	3,200	19.5
Meio Oeste e Serra Catarinense	1,011	6.1
Grande Oeste	648	3.9
Greater Florianópolis	4,119	25.1
Foz do Rio Itajaí	3,124	19.0
Alto Vale do Itajaí	1,868	11.6

a) Variables with data missing on the system.

**Figure 1:**
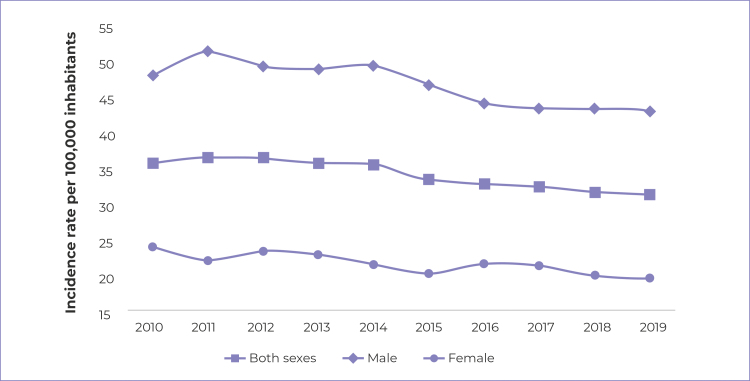
- Time series of standardized tuberculosis incidence rates by sex, Santa Catarina, 2010-2019

In all years of the period analyzed, higher tuberculosis incidence rates were found among males: in 2011, they accounted for the highest standardized rate of the time series, with 51.7 cases per 100,000 inhabitants. Among females, the highest rate was found in 2010 (24.1/100,000 inhab.), while the lowest was recorded in the last year of the period, 2019 (19.6/100,000 inhab.) (Figure 1).

We found a statistically significant decrease in the tuberculosis incidence rate in females (APC: -1.92%; 95%CI -2.63;-1.20), males (APC: 1.86%; 95%CI -2.68;-1.03) and for both sexes (APC: -1.77%; 95%CI -2.37;-1.17). Females had the highest APC value in the time series: -1.92% (Table 2).

**Table 2:** - Tuberculosis incidence rate temporal trend by sex, Santa Catarina, 2010-2019

Sex	APC^a^ %	95%CI^b^ Lower limit	95%CI^b^ Upper limit	p-value	Interpretation
Both sexes	-1.77	-2.37	-1.17	<0.001	Decreasing
Male	-1.86	-2.68	-1.03	0.001	Decreasing
Female	-1.92	-2.63	-1.20	<0.001	Decreasing

a) APC: Annual percent change; b) 95%CI: 95% confidence interval.

## DISCUSSION

The majority of tuberculosis cases occurred in males, in individuals aged 20 to 49 years, in Greater Florianopolis and in urban areas. The disease was proportionately higher among people with incomplete elementary school education and most cases had pulmonary involvement. Rates were higher among males and the incidence rates showed a decreasing trend in the period for both sexes.

Tuberculosis incidence differs between Brazil’s five geographic regions, which can make it difficult to control the disease in the country.^11^ In the same period, states with a similar population size to Santa Catarina, such as, Maranhão and Goiás, recorded around 19,000 and 9,000 cases, respectively, with Goiás standing out as having a considerably lower number than that found in this study.^12^ These differences may be related to sociocultural and economic issues, organization of health services and the carrying out of tuberculosis program actions.^11^

Pulmonary tuberculosis was predominant among the clinical forms of the disease, corroborating the results of other researchers.^13^ ^-^ ^15^ Two cross-sectional studies conducted in Brazilian university hospitals found a predominance of the pulmonary clinical form, with prevalence rates of 62.1% and 75.6%.^13^ ^,^ ^15^ An ecological study conducted in Natal, the capital city of the state of Rio Grande do Norte, also found higher prevalence of the pulmonary clinical form of the disease, although without bacteriological or histological confirmation.^14^ The result found in our study was expected, as lungs have appropriate conditions for bacterial growth and are the entry point for *Mycobacterium tuberculosis*. Therefore, the spread of the disease could possibly have occurred due to contiguity, lymphatic or hematogenous routes, especially in immunosuppressed individuals.^16^ ^,^ ^17^

Most cases were found in males and in people in the 20 to 49 age group. This profile has also been identified in other studies.^13^ ^,^ ^14^ Several factors may explain higher diagnosis of the disease in males, including biological, sociocultural, behavioral and occupational reasons.^18^ ^,^ ^19^ In some cultures, authors point out that men may travel more frequently, have more social contacts, spend more time in environments conducive to transmission, and have professions at risk for the disease.^18^ In addition, other risk factors for tuberculosis infection, such as smoking and drinking, tend to be more prevalent in males.^18^

Higher case incidence was also found in individuals with incomplete elementary schooling, corroborating the relationship between low education and tuberculosis.^20^ Tuberculosis is considered to be a serious social problem, with low schooling and poverty being important risk factors for the disease: individuals in these conditions are more predisposed to poor self-care and find it more difficult to access health services.^20^ ^,^ ^4^

Moreira et al. and Pedro & Oliveira have shown that becoming ill with tuberculosis is directly related to the precariousness of urban infrastructure and basic health services, high population density, inadequate nutrition and abuse of illicit substances.^21^ ^,^ ^22^ In addition, absence of information, which may be linked to low schooling, increases the vulnerability of population groups to the disease.^21^

Regarding the number of new cases according to Santa Catarina health macro-regions, the highest concentration was observed in Greater Florianópolis, one of the macro-regions with the highest demographic density in the state,^23^ followed by those that include municipalities on or near the coast, corroborating previous findings that indicate higher risk of developing tuberculosis in these macro-regions.^24^ The heterogeneous distribution of tuberculosis cases can be explained by different factors, among them the difference in quality of Primary Health Care services in each macro-region.^25^ Another possible explanation could be related to the fact that, generally, regions with higher population density have slums and greater population dynamics, which favors transmission of the disease. Other authors have confirmed the findings of the present study by finding spatial autocorrelation of tuberculosis with concentration in urban conglomerates of Santa Catarina’s largest cities.^26^

In this study we found a decrease in tuberculosis incidence rate in the state of Santa Catarina in both sexes. A study that analyzed the tuberculosis incidence trend in adults in all Brazilian states, between 2001 and 2017, identified a similar decreasing pattern in Santa Catarina, but with a steeper fall of 5.6% per year.^27^ Another study conducted in Santa Catarina, based on data from notifications made between 2002 and 2009, also found a drop in new cases, attributing this finding to improved access and quality of health services offered.^7^ This reduction can also be justified by the progressive improvement in health care, such as the increase in the coverage of the Family Health Strategy in Brazil, together with up to 90% directly observed treatment (DOT).^28^

In recent years, tuberculosis control strategies in Santa Catarina have been strengthened, with consequent reduction in transmission and occurrence of new cases.^29^ Standing out among these strategies is the maintenance of high coverage rates of Bacillus Calmette-Guérin (BCG) vaccination in the first year of life, early diagnosis, as well as the implementation of supervised treatment of persons with tuberculosis. Moreover, during the period analyzed Santa Catarina continued to be one of the least unequal states in Brazil, according to the summarized social indicators of the Brazilian population.^9^ However, the need still exists to consolidate effective actions to fight the disease in priority populations, as defined by the National Tuberculosis Program, such as: homeless people, populations deprived of liberty, indigenous people and people living with HIV/AIDS.^29^ To combat tuberculosis, it is necessary to invest in intersectoral articulation, especially in public policies to address the social determination of the disease.

It should be noted that the use of secondary data can be considered a limitation, in the sense of the possible occurrence of shortcomings in filling out forms and/or underreporting of the disease. However, an investigation the purpose of which was to describe the completeness of tuberculosis case records in Santa Catarina, from 2007 to 2016, concluded that the data were adequate for guiding actions to prevent and control the disease.^30^

We conclude that in the decade analyzed, there was a significant reduction in tuberculosis incidence rates in Santa Catarina, in both sexes. The profile of the cases is predominantly male, affecting individuals of economically active age and with low schooling. The most common clinical form was pulmonary, and the majority of cases were located in the Greater Florianópolis area.
